# Tunable Fabry-Pérot interferometer based on nanoporous anodic alumina for optical biosensing purposes

**DOI:** 10.1186/1556-276X-7-370

**Published:** 2012-07-03

**Authors:** Abel Santos, Victor S Balderrama, María Alba, Pilar Formentín, Josep Ferré-Borrull, Josep Pallarès, Lluís F Marsal

**Affiliations:** 1 Departament d’Enginyeria Electrònica, Elèctrica i Automàtica, Universitat Rovira i Virgili, Avda. Països Catalans 26, Tarragona, 43007, Spain

**Keywords:** Reflection, Mild anodization, Porous alumina, Pore widening, Geometric characteristics, Barcodes, 78 optical properties, Condensed-matter spectroscopy and other interactions of radiation and particles with condensed matter, 78.67.Rb nanoporous materials, 82.45.Cc anodic films

## Abstract

Here, we present a systematic study about the effect of the pore length and its diameter on the specular reflection in nanoporous anodic alumina. As we demonstrate, the specular reflection can be controlled at will by structural tuning (i.e., by designing the pore geometry). This makes it possible to produce a wide range of Fabry-Pérot interferometers based on nanoporous anodic alumina, which are envisaged for developing smart and accurate optical sensors in such research fields as biotechnology and medicine. Additionally, to systematize the responsiveness to external changes in optical sensors based on nanoporous anodic alumina, we put forward a barcode system based on the oscillations in the specular reflection.

## Background

Specular reflection (*R*_*specular*_) is the optical property defined as the mirror-like reflection of light/photons from a surface, in which light/photons from a given incoming direction are reflected into a single outgoing direction. Nanoporous structures with controlled geometrical characteristics have demonstrated to be excellent materials for developing such optical devices as resonators and microcavities [1-3]. So far, porous silicon (PSi) and nanoporous anodic alumina (NAA) have been successfully used as platforms for fabricating those optical devices [4-6]. Concretely, the physical and chemical properties of NAA (e.g., biocompatibility, thermal stability, environmental resistance, biodegradability, well-controlled geometry, and so on) make it an excellent material for developing optical biosensors. One of the foremost properties of NAA is that the pore geometry (i.e., cylindrical geometry) can be exquisitely controlled by means of the anodization parameters (i.e., acid electrolyte, anodization voltage, and anodization time). This allows us to change the effective medium by designing the pore geometry and, thus, to control the specular reflection at will.

Under certain conditions, the *R*_*specular*_ spectrum of NAA presents oscillations generated by the Fabry-Pérot effect [7]. The number, position, and intensity of these oscillations rely on the NAA thickness (i.e., the pore length (*L*_*p*_)) and its porosity (i.e., the pore diameter (*d*_*p*_)). Therefore, this property offers us an excellent opportunity for designing NAA structures with tunable optical properties by modifying the pore geometry. So far, several works have proposed a systematic and objective system for classifying certain optical properties of some nanostructures (e.g., PSi colloids, PSi nanowires, PSi particles, silica nanotubes, and so forth) [8-11]. Recently, we presented a barcode system based on the photoluminescence of NAA and its use for detecting biological substances was demonstrated [12]. Herein, we put forward an innovative barcode system based on the specular reflection of NAA. In this system, an exclusive barcode is related to each set of pore length-pore diameter by means of its *R*_*specular*_ spectrum. In this way, a wide range of unique barcodes can be generated in the UV-visible region. This barcode system is a useful method for estimating qualitatively and quantitatively the responsiveness of optical biosensors based on NAA to changes in the effective medium generated by external biological substances.

Furthermore, the pore walls in NAA can be functionalized with many materials (e.g., metals, oxides, polymers, etc.) in an accurate manner by such techniques as atomic layer deposition, dip coating, and layer-by-layer deposition. This spreads the use of NAA towards more excellent applications as selective separators, optical biosensors, and so forth.

## Methods

NAA samples were fabricated by the two-step anodization process [13]. Before anodizing, commercial aluminum (Al) substrates were electropolished in a mixture of ethanol (EtOH) and perchloric acid (HClO_4_) 4:1 (*v*:*v*) at 20 V and 5 °C. After this, the first anodization step was performed in an aqueous solution of oxalic acid (H_2_C_2_O_4_) 0.3 M at 40 V and 6 °C for 20 h. Subsequently, the alumina film was selectively dissolved by wet chemical etching in a mixture of phosphoric acid (H_3_PO_4_) 0.4 M and chromic acid (H_2_CrO_7_) 0.2 M at 70 °C. Then, the second anodization step was conducted under the same anodization conditions as the first step. The anodization time during this step was adjusted in order to modify the pore length (i.e., 60, 105, and 150 min). Finally, the pore diameter was enlarged by a wet chemical etching in an aqueous solution of H_3_PO_4_ 5 wt.% at 35 °C.

NAA samples were characterized by an environmental scanning electron microscope (ESEM FEI Quanta 600, Hillsboro, Oregon, USA. The specular reflection measurements were performed in a UV-visible spectrophotometer from Perkin Elmer (Waltham, MA, USA) with a tungsten lamp used as excitation light source at room temperature and the incoming direction of light was 15°. The standard image processing package (ImageJ, public domain program developed at the RSB of the NIH, USA) was used to carry out the ESEM image analysis [14].

## Results and discussion

**Figure 1:**
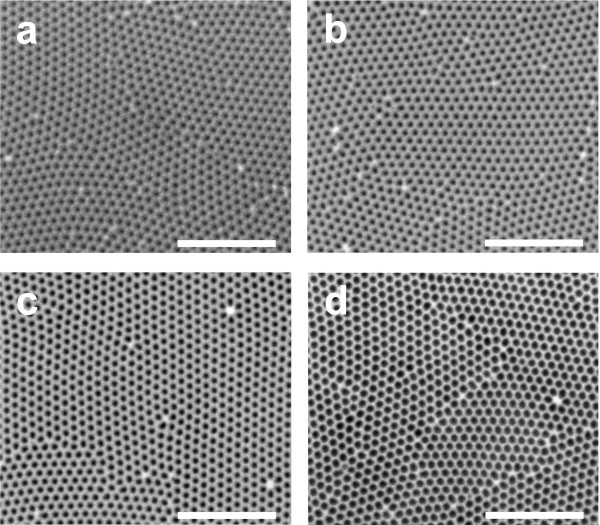
**Set of ESEM top view images of NAA samples (scale bar = 1 μm).** (**a**) R-OSC(1) (*L*_*p*_ = 5 μm, and *d*_*p*_ = 30 nm). (**b**) R-OSC(2) (*L*_*p*_ = 5 μm, and *d*_*p*_ = 41 nm). (**c**) R-OSC(3) (*L*_*p*_ = 5 μm, and *d*_*p*_ = 52 nm). (**d**) R-OSC(4) (*L*_*p*_ = 5 μm, and *d*_*p*_ = 71 nm).

**Figure 2:**
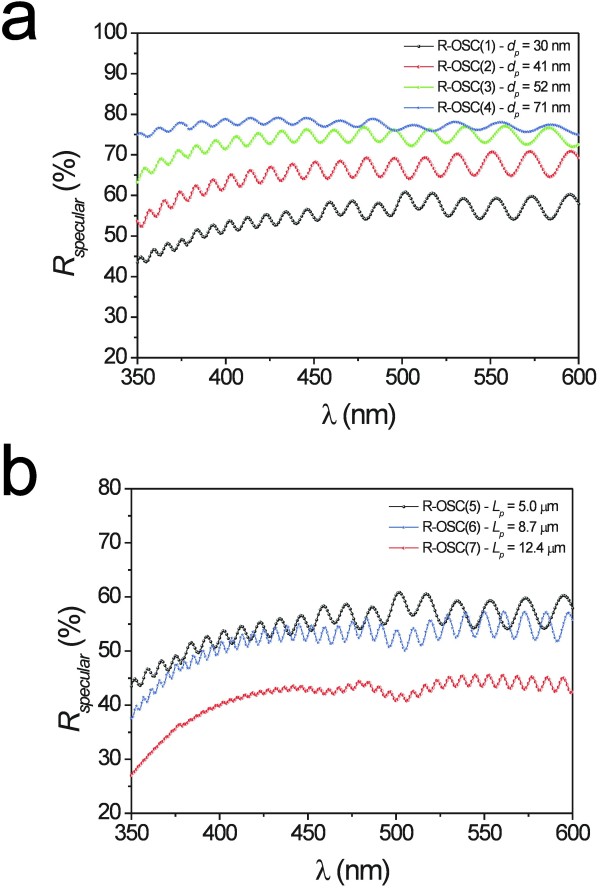
***R***_***specular***_**spectrum of NAA samples.** (**a**) R-OSC(1 to 4) (*L*_*p*_ = 5 μm, and *d*_*p*_ = 30, 41, 52, and 71 nm, respectively). (**b**) R-OSC(5 to 7) (*d*_*p*_ = 30 nm, and *L*_*p*_ = 5, 8.7, and 12.4 μm, respectively).

To study the effect of the pore geometry on the *R*_*specular*_ oscillations, the pore length and its diameter were widely modified. First, the pore length of three samples was set to three different values (i.e., 5.0, 8.7, and 12.4 μm). Then, the pore diameter of four samples with the same pore length (i.e., 5 μm) was set to four different values (i.e., 30, 41, 52, and 71 nm). So, a total of seven different NAA samples were fabricated and analyzed. Notice that, although the samples R-OSC(1) and R-OSC(5) have the same geometric characteristics, they are different samples. Figure 1 shows a set of top view ESEM images of four NAA samples with different pore diameters and the same length. The results obtained from this image analysis are summarized in Table 1. Figure 2a shows the *R*_*specular*_ spectra as a function of the pore diameter. At first glance, it is verified that the wider the pore, the higher the reflection intensity. Furthermore, it is observed that the number of peaks decreases as the pore diameter is enlarged. Another result that is worth noting is that, as Figure 2b shows, the larger the pore the lower the reflection intensity, and the number of peaks increases with the pore length.

**Table 1:** **Geometric characteristics of the NAA samples (interpore distance (*d***_***int***_) and porosity (P) -*P **= Π/(2√3))·(*d***_***p***_**/*d***_***int***_**)**^**2**^**)**

**Label**	***L***_***p***_**(μm)**	***d***_***p***_**(nm)**	***d***_***int***_**(nm)**	P (%)*
R-OSC(1)	5.0 ± 0.1	30 ± 3	103 ± 4	8.2 ± 1.7
R-OSC(2)	5.0 ± 0.1	41 ± 3	103 ± 4	15.2 ± 2.7
R-OSC(3)	5.0 ± 0.1	52 ± 3	103 ± 4	24.5 ± 4.2
R-OSC(4)	5.0 ± 0.1	71 ± 2	103 ± 4	44.4 ± 5.5
R-OSC(5)	5.0 ± 0.1	30 ± 3	103 ± 4	8.2 ± 1.7
R-OSC(6)	8.7 ± 0.1	30 ± 3	103 ± 4	8.2 ± 1.7
R-OSC(7)	12.4 ± 0.2	30 ± 3	103 ± 4	8.2 ± 1.7

The origin of these oscillations in the specular reflection spectrum of NAA is related to a strong enhancement of reflection at these wavelengths corresponding to the optical modes of the Fabry-Pérot cavity constituted by the system air-NAA-aluminum. As a result (p) marked and narrow oscillations are generated in the *R*_*specular*_ spectrum, the number, intensity, and position of which not only can be tuned by increasing the pore length but also by modifying its diameter (i.e., by changing the effective medium).

The barcode system that we propose is based on the Universal Product Code [15]. In this system, each bar position corresponds to the wavelength of each oscillation in the *R*_*specular*_ spectrum, and the higher the oscillation intensity the wider the bar in the barcode. The width of each bar is referred to the intensity scale. It means that the maximum line width would correspond to the 100% intensity in the *R*_*specular*_ spectrum. The bar width is reduced proportionally as the oscillation intensity decreases.

So far, some optical nanostructures have been successfully used as a base for developing similar barcode systems [8-12]. However, from the biotechnological point of view, the optical encoding procedure based on NAA has some advantages over those systems:

(a) The accurate control over the pore geometry in NAA makes it possible to switch the effective medium of the Fabry-Pérot cavity at will. Therefore, the *R*_*specular*_ spectrum can be designed and tuned for multiple applications. For example, those barcodes with a high number of bars are envisaged for developing optical biosensors with a high sensitivity to small external changes. Likewise, barcodes with a low number of bars are more suitable for developing optical biosensors with a high specificity (e.g., for substances with reflection maxima at localized wavelengths).

(b) The *R*_*specular*_ spectrum of NAA remains stable throughout. Hence, it is not necessary to passivate NAA for avoiding position shifts and intensity changes of the oscillations in the course of time.

(c) The cylindrical geometry of pores in NAA allows covering the pore surface with functional materials in a controlled manner for a wide range of applications.

(d) NAA is a biocompatible, thermally stable, environment-resisting, and biodegradable material.

**Figure 3:**
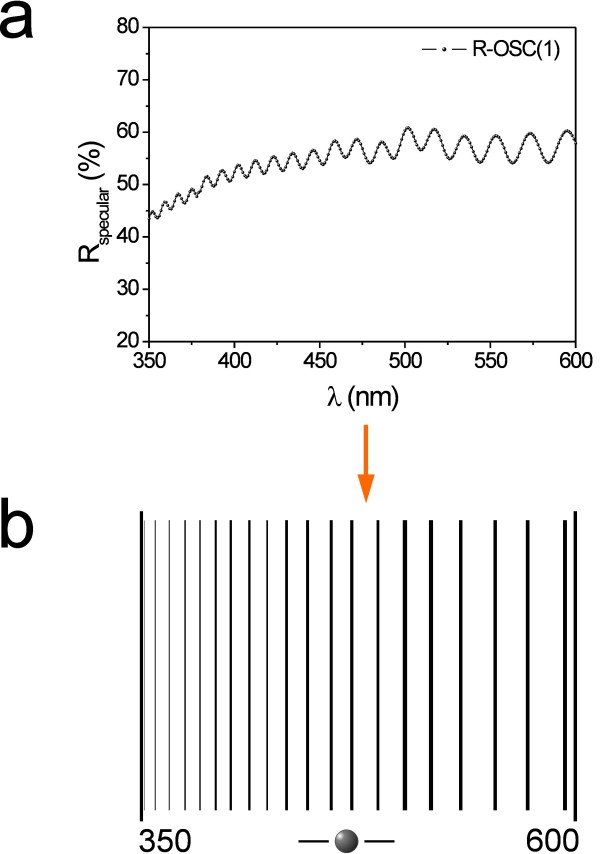
**Example of conversion of a*R***_***specular***_**spectrum into a barcode.** (**a**) *R*_*specular*_ spectrum of sample R-OSC(1). (**b**) Resulting barcode after conversion.

An example of how the *R*_*specular*_ oscillations are converted into an exclusive barcode is shown in Figure 3.

## Conclusions

In this study, we have presented an exhaustive analysis about the structural tuning of the *R*_*specular*_ oscillations in nanoporous anodic alumina. We have demonstrated that it is possible to control the *R*_*specular*_ oscillations at will by designing the pore geometry (i.e., structural engineering strategy). This is an excellent opportunity for producing structures with controlled optical properties, what is dearly useful for developing smart optical biosensors. Furthermore, we have proposed a barcode system based on the oscillations in the *R*_*specular*_ spectrum of NAA. This system is expected to be used to develop and design optical sensors in such research fields as biotechnology and medicine.

## Competing interests

The authors declare that they have no competing interests.

## Authors’ contributions

The experiments presented in this work were designed by AS and LFM. The NAAMs were fabricated by AS and VSB, characterized optically and microscopically by AS and MA. PF assisted AS and VSB during the laboratory tasks. AS, VSB, MA, PF, JFB, JP, and LFM analyzed and discussed the results obtained from the experiments. AS wrote the manuscript, and the last version of this was revised by all the authors (AS, VSB, MA, PF, JFB, JP, and LFM). All authors read and approved the final manuscript.
